# In-Situ Hydrothermal Fabrication of ZnO-Loaded GAC Nanocomposite for Efficient Rhodamine B Dye Removal via Synergistic Photocatalytic and Adsorptive Performance

**DOI:** 10.3390/nano14141234

**Published:** 2024-07-22

**Authors:** Kehinde Shola Obayomi, Sie Yon Lau, Zongli Xie, Stephen R. Gray, Jianhua Zhang

**Affiliations:** 1 Department of Chemical Engineering, Curtin University, CDT 250, Miri 98009, Sarawak, Malaysia; 2 Institute for Sustainable Industries and Liveable Cities, Victoria University, Werribee, VIC 3030, Australia; 3 Commonwealth Scientific and Industrial Research Organization (CSIRO), Private Bag 10, Clayton South, VIC 3169, Australia

**Keywords:** ZnO-based catalyst, rhodamine B, UV-light, photocatalytic degradation, adsorption

## Abstract

In this work, zinc oxide (ZnO)/granular activated carbon (GAC) composites at different ZnO concentrations (0.25M-ZnO@GAC, 0.5M-ZnO@GAC, and 0.75M-ZnO@GAC) were prepared by an in-situ hydrothermal method and demonstrated synergistic photocatalytic degradation and adsorption of rhodamine B (RhB). The thermal stability, morphological structure, elemental composition, crystallographic structure, and textural properties of developed catalysts were characterized by thermal gravimetric analysis (TGA/DTG), scanning electron microscopy equipped with energy dispersive-x-ray (SEM-EDS), X-ray diffraction (XRD), and Brunauer–Emmett–Teller (BET) analysis. The successful loading of ZnO onto GAC was confirmed by SEM-EDS and XRD analysis. The BET surface areas of GAC, 0.25M-ZnO@GAC, 0.5M-ZnO@GAC, and 0.75M-ZnO@GAC were 474 m^2^/g, 450 m^2^/g, 453 m^2^/g, and 421 m^2^/g, respectively. The decrease in GAC could be attributed to the successful loading of ZnO on the GAC surface. Notably, 0.5M-ZnO@GAC exhibited the best photocatalytic degradation efficiency of 82% and 97% under UV-A and UV-C light over 120 min, attributed to improved crystallinity and visible light absorption. The photocatalytic degradation parameters revealed that lowering the RhB concentration and raising the catalyst dosage and pH beyond the point of zero charge (PZC) would favor the RhB degradation. Photocatalytic reusability was demonstrated over five cycles. Scavenger tests revealed that the hydroxyl radicals (^•^OH), superoxide radicals (O_2_^−•^), and photoinduced hole (h^+^) radicals play a major role during the RhB degradation process. Based on the TOC results, the RhB mineralization efficiency of 79.1% was achieved by 0.5M-ZnO@GAC. Additionally, GAC exhibited a strong adsorptive performance towards RhB, with adsorption capacity and the RhB removal of 487.1 mg/g and 99.5% achieved within 90 min of equilibrium time. The adsorption characteristics were best described by pseudo-second-order kinetics, suggesting chemical adsorption. This research offers a new strategy for the development of effective photocatalyst materials with potential for wider wastewater treatment applications.

## 1. Introduction

With increasing industrial technology, population growth, and urban development, the concern for water pollution is increasing worldwide due to the release of harmful organic pollutants into the ecosystem, posing a severe threat to both human and aquatic health [1,2]. The textile industry, which is one of the major sources of organic pollutants, especially dyes, has had a noteworthy effect on the immediate environment [3]. Rhodamine B (RhB) is a cationic dye and the most toxic of the xanthene class [4]. RhB is water-soluble, a water tracer fluorescent dye with excellent photophysical structures, and the oldest synthetic dye that has found applications in several industries, especially in the food and fabric industries [5]. The discharge of colored RhB dye in wastewater has resulted in reduced oxygen levels, blocked photocatalytic activities, prevented aquatic plant growth, and produced coloration change [6]. RhB dyes are mutagenic, carcinogenic, non-biodegradable, and chemically stable. Moreover, the negative impact of dyes on human health includes skin irritation, dermatitis, and kidney, liver, eye, and reproductive system damage [7]. However, the continuous discharge and accumulation of RhB dye in waterbodies will have a severe effect on human and aquatic life and agricultural produce [8]. Therefore, in order to reduce environmental loads of this dye to a tolerable level or to remove it completely to avert impending danger and diminish pollution, there is an urgent need to treat wastewater containing RhB dye before it is discharged.

Over the past decades, researchers have explored various techniques, including membrane filtration, coagulation and flocculation, adsorption, photocatalytic degradation, ion exchange, and aerobic and anaerobic processes [3,5,9,10] to treat wastewater containing RhB. However, many of these techniques suffer from inefficiencies and drawbacks such as high cost, generation of secondary pollutants, sludge formation, poor treatment efficiency, and high energy consumption [11]. Among the techniques, adsorption and light-driven photocatalysis have emerged as the most effective due to their environmental friendliness, long-term stability, cost-effectiveness, potential to utilize solar irradiation, simple operation, and non-toxic nature [12,13]. Photocatalysis uses light irradiation to excite valence band electrons, triggering charge carriers that undergo a series of redox reactions. These reactions produce a variety of free radicals in solution that can then engage in oxidation-reduction reactions with organic contaminants as the target molecules. In addition, the adsorption process involves the use of adsorbent materials with a high surface area and a well-developed porous structure [12,14]. The adsorbent selection is vital since removing pollutants from water should be cost-effective, simple, and have competitive adsorption capacity towards the target pollutants [15].

Recent advances in nanotechnology have promoted the use of nanomaterials to address water pollution and wastewater treatment problems. Nanoparticles (NPs) with dimensions under 100 nm offer unique chemical and physical perspectives for water treatment [16]. Zinc oxide (ZnO), a semiconductor with a bandgap of 3.37 eV and high excitation energy (60 meV) properties, has attracted attention recently due to its high electromobility, strong electrochemical or thermal stability, biocompatibility, and non-toxicity nature [17]. Compared with other metallic oxides, zinc oxide has the unique capacity to exhibit both physical and chemical behavior due to its tunability [18]. Additionally, ZnO nanoparticles (ZnO NPs) demonstrate good practicality as a photocatalyst and adsorbent for the efficient degradation of dyes in wastewater due to their robust chemical stability, superior optical qualities, electrochemical capabilities, and excellent adsorption capacity [19]. These characteristics are strongly influenced by size, shape, and structure [20]. Despite many advantages, the challenges such as retrieving and recycling after use, maintaining colloidal stability of NPs in water at high concentrations, and the rapid recombination of photogenerated holes and electrons hinder their use in wastewater treatment. The development of techniques for enhancing ZnO hybrid materials to satisfy environmental and energy constraints is, therefore, highly desirable [21]. However, the photocatalytic activity of ZnO is hindered by the ultraviolet light spectrum due to its poor quantum yield and broad band gap energy (3.37 eV), which also promotes electron–hole recombination. To improve ZnO photocatalytic activity and oxidation process, the latter should be prevented from producing electron/hole pairs, with the excited electron reacting with a pollutant [22]. Therefore, to overcome these challenges and improve the photocatalytic activity of ZnO, there is a need to support ZnO in terms of functionalization such as loading with other materials [23].

In recent years, many researchers have expressed interest in the fabrication of more effective ZnO hybrid materials for wastewater treatment by loading them on other materials, including zeolites, clays, metal-organic framework (MOF), carbon-based materials, and metal oxides [24,25,26,27,28]. Prior studies have demonstrated that loading ZnO on activated carbon (AC) enhanced adsorption capacity because the resultant composite material allows enhanced interaction between the composite material and the dyes due to the presence of abundant active sites [29,30,31,32]. Recently, Yu et al., [33] fabricated ZnO/biochar nanocomposites via simple ball milling with excellent photocatalytic and adsorption properties towards methylene blue (MB) under UV light with 95.19% removal efficiency. Therefore, it is necessary to develop straightforward, affordable, non-toxic, cost-effective, effective as well as reusable semiconductor nanocomposites capable of adsorbing and photodegrading RhB from aqueous media. The novelty of this work lies in the loading of different concentrations of ZnO on granular activated carbon as a support material for the treatment of RhB. Although ZnO has been the subject of investigations into photocatalytic performance, this work goes further by investigating the use of a carbonaceous (granular activated carbon) material as a support to overcome the drawbacks in water treatment. Furthermore, this study provides more insight into the performances of the developed catalysts by investigating their adsorptive-photodegradation performance towards RhB. Again, the study also compares the photodegradation ability of catalysts under UV-A and UV-C lights which has never been reported in previous studies. Finally, as a result of human activity-related water resource contamination, our work helps to develop more efficient and effective wastewater treatment solutions, which are much-needed.

In this study, we employed an in-situ hydrothermal reaction technique to load varying concentrations of ZnO onto granular activated carbon to form 0.25 ZnO@GAC, 0.5 ZnO@AC, and 0.75 ZnO@AC nanocomposites. These nanocomposites were then investigated for their combined photocatalytic-adsorptive potentials demonstrated towards RhB dye removal. The prepared nanocomposites were characterized using scanning electron microscopy (SEM) coupled with energy-dispersive X-ray spectroscopy (EDS), Brunauer–Emmett–Teller (BET), thermal gravimetric analysis/differential thermal analysis (TGA/DTA), and X-ray diffraction (XRD) to confirm the successful uniform dispersion of ZnO on the AC surface.

## 2. Materials and Methods

### 2.1. Materials

Analytical-grade chemicals and materials, including zinc acetate dihydrate (C_4_H_10_O _6_Zn), rhodamine B (RhB), granular activated carbon (GAC), calcium persulfate (PS), isopropanol (IPA), triethanolamine (TEOA), and benzoquinone (BQ were purchased from Sigma-Aldrich chemicals, Australia.

### 2.2. Synthesis of ZnO-Loaded Activated Carbon (ZnO@GAC)

Briefly, different concentrations (0.25 M, 0.5 M, and 0.75 M) of zinc acetate solutions were prepared by measuring an appropriate amount of zinc acetate dihydrate into 500 mL beakers containing 200 mL deionized water and stirring continuously on a magnetic stirrer at room temperature for 30 min to achieve homogeneity. Thereafter, 5 g of GAC was added to each beaker containing the homogeneous solution under continuous stirring for another 2 h. Finally, the resultant precipitate was collected by filtration, washed several times with deionized water to neutralize, and then oven-dried at 80 °C for 15 h. Finally, the dried solids were then calcined in a muffle furnace at 400 °C for 2 h, and the as-synthesized nanocomposites were grounded, stored in airtight plastic bags, and labeled as 0.25M-ZnO@GAC, 0.5M-ZnO@GAC, and 0.75M-ZnO@GAC nanocomposites.

### 2.3. Characterization

The prepared nanocomposites were characterized using scanning electron microscopy coupled with energy-dispersive X-ray spectroscopy (SEM-EDS, VE-9800, Keyence, Osaka, Japan) to examine the surface morphology and elemental composition. Brunauer–Emmett–Teller (BET-BEL, BELSORP MINI II, Japan) was utilized to measure the nanocomposites’ pore volume, pore size, and surface areas using nitrogen adsorption-desorption at 77 K. X-ray diffraction techniques (XRD; Philips, PW1730, Netherlands) investigated the nanocomposite’s crystal structures. The XRD patterns were obtained using a D8 Advance Bruker X-ray diffractometer (Cu Kα radiation, 40 kV/40 mA) with a step size of 0.05 and a scanned range of 2ϴ = 20° to 70°. The thermal property of nanocomposites was investigated in the temperature range of 25 °C to 800 °C at a heating rate of 10 °C/min in air using the thermogravimetric-differential thermogravimetry analysis (TGA-DTG) (TA, Q600, USA).

### 2.4. Photocatalytic Degradation Test

The photocatalytic activity of the prepared nanocomposites towards RhB degradation under the influence of light was observed through spectra changes obtained through UV-VIS spectroscopy. Typically, 50 mg each of GAC, 0.25M-ZnO@GAC, 0.5M-ZnO@GAC, and 0.75 M-ZnO@GAC were measured and transferred into a set of 1000 mL beakers containing 500 mL of RhB solution (5 mg/L), and the solution initial pH was maintained. The solution mixtures were then placed on a magnetic stirrer and stirred continuously at room temperature for 30 min in the dark prior to light exposure to achieve equilibrium adsorption-desorption between the RhB and the nanocomposites. After establishing equilibrium in the dark, the mixtures were exposed to UV-A and UV-C (6 UV lamps) lights of wavelengths 352 nm and 254 nm for 120 min. The distance between the light and the beaker was 20 cm, with irradiance of 20–25 W/m^2^. During the degradation process, samples were taken at intervals of 15 min and filtered through 0.45 µm polymeric membranes to separate the nanocomposites from the filtrates. The residual concentrations of RhB after degradation were measured using the UV-VIS spectrometer (Shimadzu UV-2450, Tokyo, Japan) at a wavelength of 554 nm. The RhB degradation efficiency was calculated using the equation:

(1) $$\mathrm{Degradation}\;(\%)=\frac{C_{0}-C_{t}}{C_{0}}\times 100$$

where $C_{0}$ and $C_{t}$ are the initial and final (at time, t) concentrations before and the after degradation of RhB. Furthermore, the total organic carbon (TOC) test was employed to measure the degree of mineralization using a TOC analyzer (Shimadzu, TOC-VCPN, Japan). The TOC removal was calculated using the equation:

(2) $$\mathrm{TOC}\;\mathrm{removal}\;(\%)=\frac{TOC_{0}-TOC_{t}}{TOC_{0}}\times 100,$$

where ${TOC}_{0}andTOC_{t}$ are the initial and at a time t, TOC concentrations.

### 2.5. Batch Adsorption Test

The adsorptive performances of GAC, 0.25M-ZnO@GAC, 0.5M-ZnO@GAC, and 0.75M-ZnO@GAC towards RhB were measured in a batch system. Briefly, 50 mg of GAC, 0.25M-ZnO@GAC, 0.5M-ZnO@GAC, and 0.75 M-ZnO@GAC were placed into a set of 1000 mL beakers containing 500 mL of RhB (50 mg/L), and the initial pH solution of the mixture was maintained. The mixtures were placed on a magnetic stirrer and stirred continuously for 120 min at room temperature while maintaining the pH of the solution. Prior to attaining equilibrium, samples were taken at different time intervals, filtered, and measured to determine the residual concentrations after adsorption using the UV-Vis spectrometer at a wavelength of 554 nm. The RhB percentage removal was calculated using the equation:

(3) $$\mathrm{Removal}\;(\%)=\frac{C_{0}-C_{t}}{C_{0}}\times 100$$

### 2.6. Stability Test

In this study, the stability of the developed catalyst was investigated after the photocatalytic degradation study. The developed catalyst was washed with ethanol and deionized water several times to remove RhB from the catalyst surface. The washed catalyst was centrifuged, dried in an oven at 80 °C for 3 h, and reused in several photocatalytic degradation cycles.

## 3. Results and Discussion

### 3.1. Characterization

#### 3.1.1. XRD Analysis

**Figure 1:**
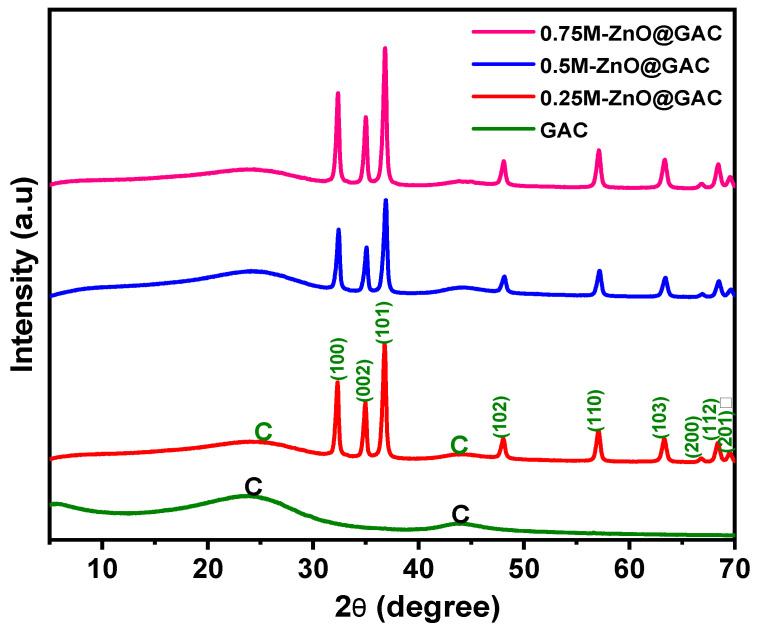
XRD diffraction patterns for GAC, 0.25M-ZnO-GAC, 0.5M-ZnO-GAC, and 0.75M-ZnO-GAC.

The structure of the developed composite materials and the formation of ZnO in the GAC were examined using the XRD patterns, recorded over a 2θ range of 5–70°. As shown in Figure 1, the GAC diffraction pattern shows two broad peaks at 24.5° and 43.9°, corresponding to the (002) and (100) planes of graphitic carbon (JCPDS card no. 41–1487). The broadness of the peaks indicates the amorphous nature of the GAC material [34]. The crystallinity diffraction pattern of hexagonal-wurtzite-structured ZnO with characteristic peaks (32.3°, 34.9°, 36.7°, 48.0°, 57.1°, 63.2°, 66.82°, 68.4°, and 69.6°) relating to (100), (002), (101), (102), (110), (103), (200), (112), and (201) were present in the spectra of 0.25M-ZnO@GAC, 0.5M-ZnO@GAC, and 0.75M-ZnO@GAC composites, suggesting that well-crystalized ZnO was loaded successfully on the GAC [35,36]. The ZnO sharp peaks in the developed composite materials are in good agreement with JPCDS card no. 36-1451, suggesting the formation of highly purified ZnO crystals [37].

#### 3.1.2. BET Measurement

The BET measurement analysis examined the surface area and porosity of the developed GAC and ZnO-based nanocomposites. The N_2_ adsorption-desorption isotherm plots of the prepared ZnO-based nanocomposites are depicted in Figure S1a–d. The plots revealed clearly that the materials exhibited type IV isotherm characteristics with prominent H4 hysteresis loop at high relative pressure (P/P_0_) ranging from 0–0.1. This fact suggests that the ZnO-based nanocomposites comprise microporous and mesoporous structures [38]. The textural properties presented in Table 1 show clearly that the BET and Langmuir surface area (S_BET_ and S_Lang_) follow the trend of GAC (481 and 718 m^2^/g) > 0.5M-ZnO@GAC (453 and 679 m^2^/g), 0.25M-ZnO@GAC (450 and 675 m^2^/g), and 0.75M-ZnO@GAC (421 and 632 m^2^/g). The decrease in ZnO-based nanocomposites’ surface area when compared with GAC could be attributed to pore blockage during the loading of ZnO. Furthermore, the surfaces of the developed materials show the presence of micropores and mesopores but are majorly dominated by mesopores (2–50 nm), suggesting that ZnO-based nanocomposites significantly favor the adsorption-photocatalytic degradation of pollutants [39]. The formation of mesoporous structure could result from the decomposition of zinc acetate and the formation of gases during thermal treatment [40]. 

#### 3.1.3. SEM/EDX Analysis

**Figure 2:**
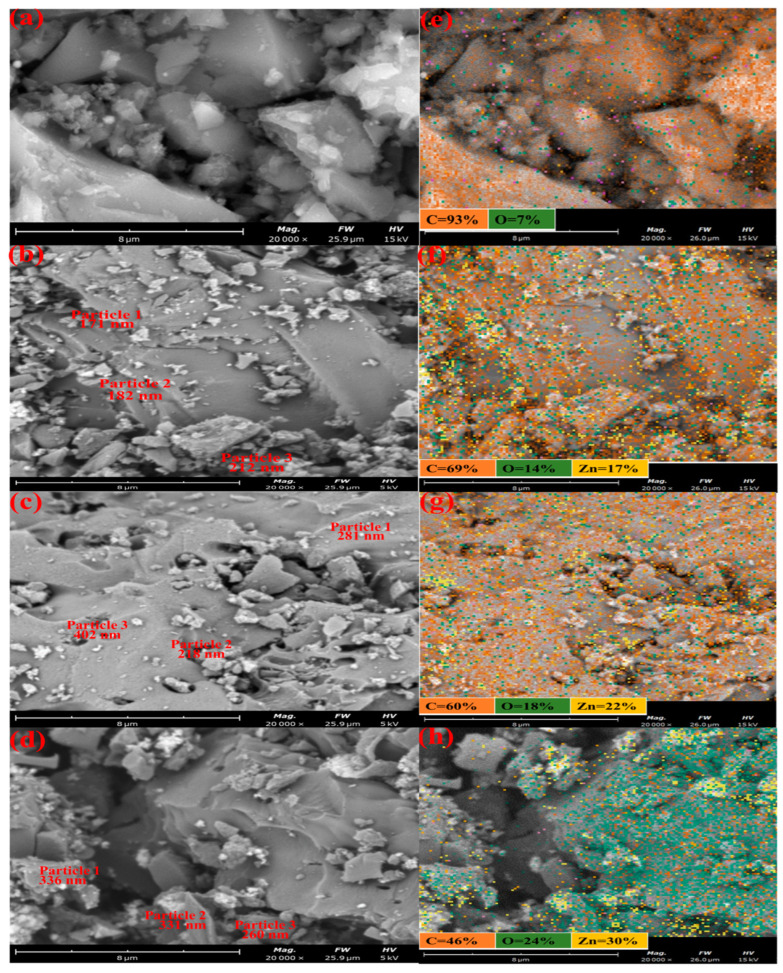
SEM micrographs and EDX analysis for (**a**,**b**) GAC, (**c**,**d**) 0.25M-ZnO@GAC, (**e**,**f**) 0.5M-ZnO@GAC, and (**g**,**h**) 0.75M-ZnO@GAC.

The surface texture and morphology of GAC and GAC-ZnO-based nanocomposites were investigated using the SEM analysis and the results are presented in Figure 2. The SEM micrograph and EDS mapping of GAC are shown in Figure 2a,e. The images revealed that the GAC surface was heterogeneous, rough, and less porous with crevices, and holes of different sizes. The hole formation on the GAC surface could be attributed to the loss of volatile components such as carbon in the form of CO and CO_2_ [41]. The EDS mapping revealed that the GAC surface is majorly dominated by carbon, followed by oxygen. The SEM images and EDS mapping of 0.25M-ZnO@GAC, 0.5M-ZnO@GAC, and 0.75M-ZnO@GAC presented in Figure 2b–d revealed the presence of agglomerated ZnO particles, smooth, more porous, and fewer cracks on the surfaces. It can be observed that the loaded ZnO particle (identified by spot EDS in Table 2) sizes measured in the SEM are in the range of 172–402 nm and tend to be smaller when less ZnO is loaded. The EDS shows that more ZnO was loaded on the AC. The decrease in the pores of 0.75M-ZnO@GAC was attributed to the formation of more ZnO, resulting in the blockage of the pores [42]. The EDS mapping depicted in Figure 2f,g, revealed that the surfaces of 0.25M-ZnO@GAC, 0.5M-ZnO@GAC, and 0.75M-ZnO@GAC contain mostly carbon, zinc, and oxygen. Furthermore, the presence of ZnO was observed to be more on the 0.75M-ZnO@GAC surface, suggesting that the ZnO increases with increasing concentration [43]. Furthermore, the carbon amount was high in all the materials but decreased as more ZnO was loaded onto the GAC (Table 2). The presence of Zn revealed that the ZnO was successfully loaded on the GAC surface [27]. The SEM images at different magnifications were presented in Figure S2. Further, the elemental compositions of three different particles were observed and presented in Table 2. It was observed from the results that as the ZnO concentrations were increased, the percentage weight of carbon decreased, while that of oxygen and zinc increased.

#### 3.1.4. TGA/DTG Analysis

**Figure 3:**
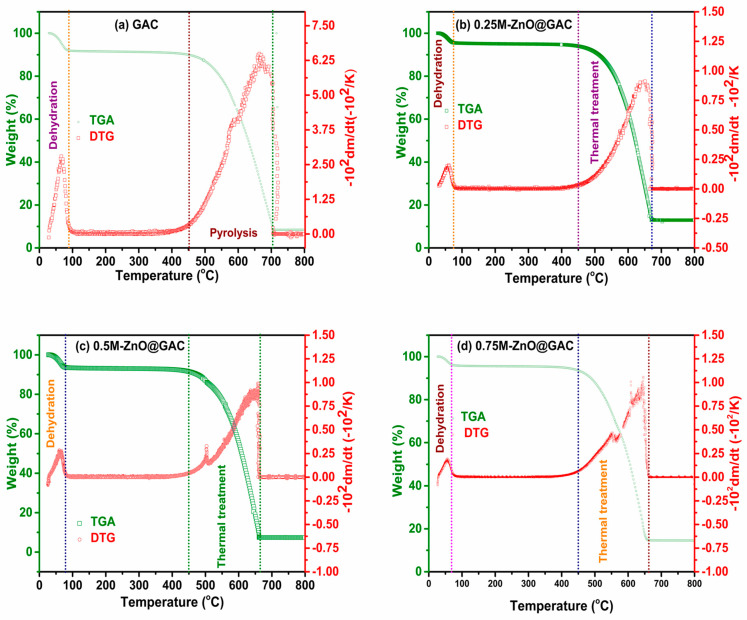
TGA/DTG analysis of (**a**) GAC, (**b**) 0.25M-ZnO@GAC, (**c**) 0.5M-ZnO@GAC, and (**d**) 0.75M-ZnO@GAC.

The thermal stabilities of GAC and ZnO@GAC composites were examined using thermal gravimetry (TGA) and derivative thermogravimetric analysis (DTG), with results shown in Figure 3. The TGA and DTG analysis of GAC as seen in Figure 3a clearly revealed two stages of weight loss at 87 °C and 450 ^o^C. In the first stage, the weight loss of about 8.0% between 25 and 87 °C was attributed to adsorbed water molecule dehydration [44]. However, no significant weight loss (2.04%) between 87 and 450 °C occurred. A noticeable weight loss of about 83.6% during the second stage between 450 and 700 °C could be attributed to the decomposition of hemicellulose, cellulose, and volatile matter [45,46]. The GAC material was thermally stable above 700 °C. As observed, the DTG analysis revealed that GAC’s sharpest weight loss peak was at 664 ^o^C. Similarly, the TGA pattern of 0.25M-ZnO@GAC, 0.5M-ZnO@GAC, and 0.75M-ZnO@GAC, as shown in Figure 3b–d, also revealed two stages of weight loss. In the first stage, weight loss of 4.5%, 6.2%, and 3.86% was observed for 0.25M-ZnO@GAC (25-77 ^o^C), 0.5M-ZnO@GAC (25–76 °C), and 0.75M-ZnO@GAC (25–71 °C). The weight loss at the first stage could be attributed to the evaporation of water molecules during the thermal treatment [29]. Furthermore, the significant amount of weight loss between 450 ^o^C and 670 °C (82.5%), 450 °C and 660 °C (86.1%), and 450 °C and 658 °C (82.3%) could be assigned to zinc acetate decomposition during the thermal process. The developed 0.25M-ZnO@GAC, 0.5M-ZnO@GAC, and 0.75M-ZnO@GAC showed thermal stability between 670–800 °C, 660–800 °C, and 658–800 °C, respectively. The DTG curve revealed the highest weight loss peaks for 0.25M-ZnO@GAC, 0.5M-ZnO@GAC, and 0.75M-ZnO@GAC at 651 °C, 650 °C, and 645 °C, respectively.

### 3.2. Photocatalytic Performance

The photocatalytic performance of GAC and ZnO-based GAC (0.25M-ZnO@GAC, 0.5M-ZnO@GAC, and 0.75M-ZnO@GAC) towards RhB at different irradiation times (0–120 min) were investigated using both the UV-A and UV-C lights, as depicted in Figures S3a–e and S4a–e, respectively. The characteristics absorbance peak of RhB was measured at λ = 553 nm. Initially, the nanocomposite materials, including the RhB, were subjected to equilibrium adsorption for 30 min to ensure that the GAC and ZnO@GAC materials adsorbed adequate RhB that would allow enough contact area between the RhB and the developed materials [47]. However, under UV-lights (UV-A and UV-C), the results presented in Figures S3a and S4a demonstrated that the photodegradation of RhB in the absence of photocatalyst showed no significant changes in the absorbance spectra during the irradiation process, suggesting the RhB’s stability under UV-light irradiation [48]. However, notable changes were observed in the absorbance peaks of GAC towards RhB during the photocatalytic degradation process. The changes in the absorbance peaks of GAC at different irradiation times were attributed to RhB adsorption because the GAC has no catalyst and photodegradation ability depends on the photocatalyst mechanisms [49,50]. Furthermore, the photocatalytic performance of 0.25M-ZnO@GAC, 0.5M-ZnO@GAC, and 0.75M-ZnO@GAC towards RhB in the presence of UV-A Figure S3b–e and UV-C Figure S4b–e lights revealed a significant change in the absorbance spectra, suggesting that the presence of ZnO at different concentrations greatly influenced the photocatalytic performance towards RhB. The results also indicate that the GAC-ZnO-based composites’ absorption edges are greatly enhanced by the ZnO loading, thereby increasing their capacities to absorb UV light [51]. Notably, the photocatalytic performances of 0.25M-ZnO@GAC, 0.5M-ZnO@GAC, and 0.75M-ZnO@GAC towards RhB under the influence of UV-C light show higher photocatalytic activity, with the RhB degradation rates almost becoming straight lines after 120 min. Moreover, the photosensitization ability of GAC, 0.25M-ZnO@GAC, 0.5M-ZnO@GAC, and 0.75M-ZnO@GAC can be explained based on the differences in their photodegradation performances towards RhB under UV-A and UV-C by comparing their absorption spectrums Figures S3a–e and S4a–e. The spectrum revealed that the photodegradation of RhB under UV-C outperformed that of UV-A, and this could be attributed to the following reasons; first, the UV-C releases more energy than the UV-A leading to more electron excitation; secondly, the UV-A absorption wavelength (315–400 nm) during photocatalytic activity did not overlap with the adsorption spectra of TC (356 nm), resulting in minimal degradation rate; and thirdly, the UV-C absorption wavelength of 254 nm will overlap the absorption wavelength of TC, leading to the formation of an excited TC by a mechanism of photosensitization and a higher formation rate of highly oxidized radicals on the catalyst surfaces [52].

**Figure 4:**
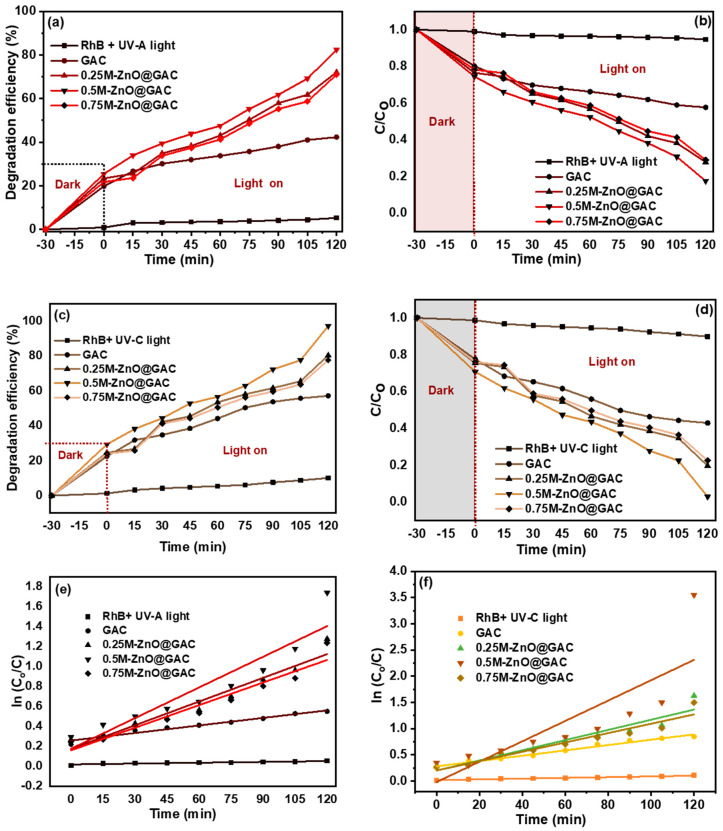
Effect of contact time on (**a**,**b**) photocatalytic degradation under UV-A light, (**c**,**d**) photocatalytic degradation under UV-C light and (**e**,**f**) photocatalytic degradation kinetics under UV-A and UV-C lights for RhB, GAC, 0.25M-ZnO-GAC, 0.5M-ZnO-GAC, and 0.75M-ZnO-GAC (initial RhB pH, initial concentration= 5 mg/L and temperature =298 K).

The effect of contact time on the photocatalytic degradation of RHB was investigated using the developed catalysts and the results are depicted in Figure 4a–d. However, to ensure that adsorption equilibrium was established between the catalysts and RhB, the solution mixture was first stirred for 30 min in the dark before starting the degradation process [23]. As observed from the plots, the curves of RhB without the addition of catalysts under UV-A and UV-C light conditions were flat (stable under UV-light) without the addition of catalyst and their degradation rates were 5 and 10% after 120 min [53]. The absorbance curve of GAC also showed little degradation under the influence of both lights with 42 and 57% degradation rates. In addition, the low performance of GAC towards RhB is attributed to the absence of a catalyst. In comparison, the degradation performance of 0.25M-ZnO@GAC, 0.5M-ZnO@GAC, and 0.75M-ZnO@GAC towards RhB were 72%, 82%, and 71% under UV-A light and 80%, 97%, and 78% under UV-C lights, respectively. The results revealed that the materials loaded with ZnO catalyst demonstrated higher degradation efficiency than those without catalysts. This was attributed to the surface plasmon, which was enhanced by the photoexcited electron surface valence of the ZnO. This surface plasmon significantly improved the RhB degradation efficiency of the ZnO-based composites, thereby enhancing the utilization efficiency of the UV-A and UV-C lights [12]. Furthermore, the degradation efficiency of 0.5M-ZnO@GAC under the influence of the UV-A and UV-C lights was observed to be higher than that of 0.25M-ZnO@GAC and 0.75M-ZnO@GAC. The outstanding performance of 0.5M-ZnO@GAC’s photocatalytic activity was attributed to effective photogenerated carrier separation. However, an increase in the ZnO concentration from 0.25 M to 0.5 M led to an increase in the degradation efficiency, but a further rise in concentration (0.75 M) resulted in a decrease in the degradation rate. This could be due to an excessive amount of white ZnO on the GAC surface, which affected photon absorption [54]. Also, the loading of low ZnO concentration on GAC enhanced the 0.25M-ZnO@GAC and 0.5M-ZnO@GAC optical characteristics and lattice structure, thereby decreasing the energy band gap and absorbing produced electrons in the catalyst structure [23]. However, at higher ZnO concentrations, additional oxygen valences and Zn^2+^ interact to form new photoinduced electrons and holes, thereby decreasing the photocatalytic activity of the catalyst [55]. Additionally, the RhB degradation rate decrease could be attributed to agglomeration of the catalyst active sites due to higher ZnO concentration loading, thereby resulting in photoexcitation and the absorption of light blockage [50].

The first-order kinetic rate constant of RhB photodegradation on the developed catalysts was calculated using the Langmuir–Hinshelwood equation:

(4) $$\ln\frac{C}{C_{0}}=-kt+\ln C_{O},$$

where k, t, C_0_, and C represent the pseudo-first-order rate constant, degradation time, and concentration, respectively.

The pseudo-first-order kinetics plots depicted in Figure 4e,f for UV-A light and UV-C light were investigated to fit the photocatalytic degradation data of RhB. The photocatalytic rate constant (k_1_) was calculated from the slope of ln (C_0_/C) against time, as presented in Table 2. The results in Table 3 further revealed that the photocatalytic performance of 0.5M-ZnO@GAC under UV-C and UV-C lights exhibited the best degradation efficiency, as seen in their higher k_1_ values (0.010 min^−1^ and 0.019 min^−1^), suggesting that the 0.5M ZnO loaded GAC exhibited enhanced photodegradation [56,57]. 

**Figure 5:**
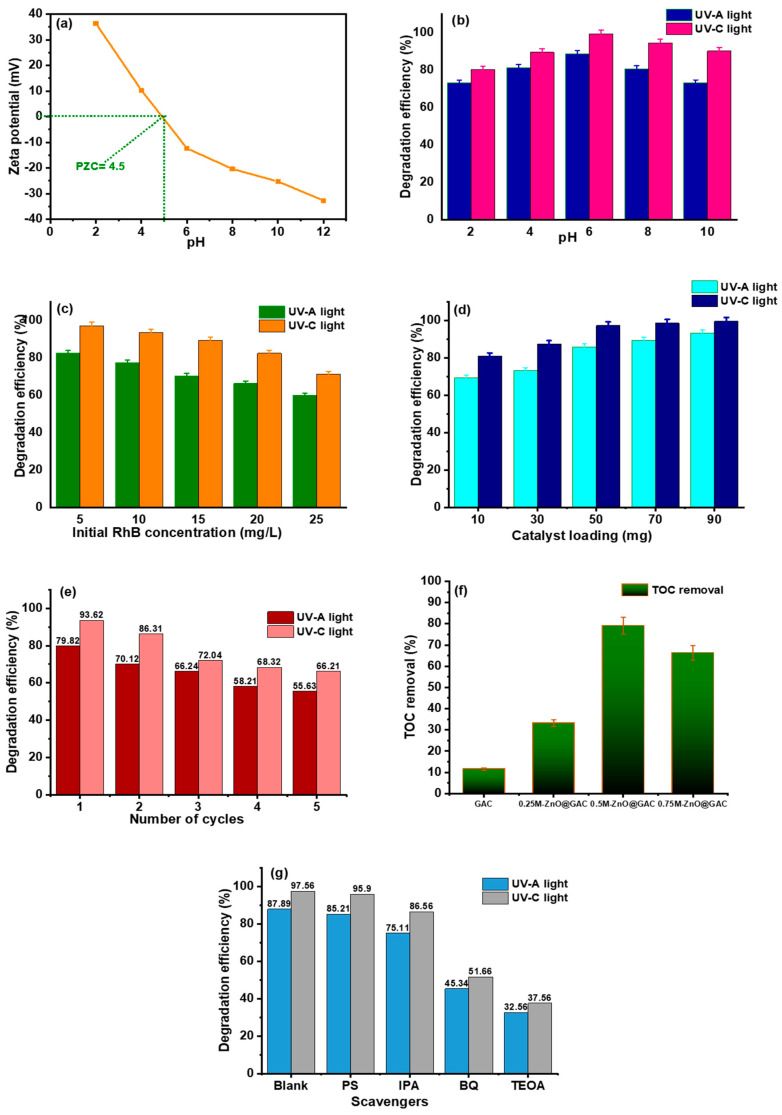
Plots of (**a**) point of zero charge (PZC), (**b**) effect of pH, (**c**) initial RhB concentrations, (**d**) effect of catalyst, (**e**) reusability study, (**f**) effect of scavengers on RhB degradation towards 0.5M-ZnO@GAC, and (**g**) TOC removal.

As presented in Figure 5a, the point of zero charge (PZC) of 0.5M-ZnO@GAC is 4.5, demonstrating the point at which the charge on 0.5M-ZnO@GAC surface becomes zero. However, the 0.5M-ZnO@GAC surface becomes positively charged when the pH < PZC and negatively charged when the pH > PZC value is higher. The impact of pH was investigated on the photocatalytic degradation of RhB using 0.5M-ZnO@GAC as the selected catalyst based on the outstanding performance compared with other catalysts. The experiment was carried out at varying pH (2, 4, 6, 8, and 10) under constant catalyst dosage (50 mg), RhB concentration (5 mg/L), and irradiation time (120 min). The results depicted in Figure 5b and Table S1 showed that the degradation efficiency of 0.5M-ZnO@GAC towards RhB increased significantly from 73.27% and 80.32% at pH 2 to 88.8% and 99.3% at pH 6 under UV-A and UV-C lights, respectively. In addition, the photocatalytic rate constant (k_1_) also increased as the pH values increased from 2 to 6. The highest degradation efficiency of RhB at pH 6 could be due to the fact that under an acidic medium, the COO^−^ present in RhB is protonated; COOH and the cationic form (N^+^(C_2_H_5_)_2_) are left in the solution, thereby promoting electrostatic attraction between the positive charges of RhB and negative charges on 0.5M-ZnO@GAC surface (pH > PZC) [58]. However, as the pH increased beyond 6, a decrease in the RhB degradation efficiency and rate constant was observed. This can be ascribed to the fact that at higher pH, the RhB ionization improved, resulting in the formation of more negatively charged ions and thereby causing electrostatic repulsion between RhB and 0.5M-ZnO@GAC [23]. Furthermore, negative carboxylate functional groups of the RhB molecule may repel negatively charged hydroxyl anions present at the 0.5M-ZnO@GAC surface, resulting in the less formation of oxidizing species such as O^−^ [59].

The impact of RhB concentration on the photocatalyst activity of 0.5M-ZnO@GAC towards RhB was examined under UV-A and UV-C lights at varied initial RhB concentrations (5, 10, 15, 20, and 25 mg/L) at constant irradiation time of 120 min, pH 6, and an adsorbent dosage of 50 mg. The results in Figure 5c and Table S2 revealed that the RhB degradation and photocatalytic rate constant under UV-A and UV-C were higher at lower concentrations and decreased as the concentration increased. At 5 ppm, 90.3% and 99.2% RhB degradation were achieved under UV-A and UV-C lights, while 66.3% and 82.5% were achieved at 25 mg/L. This finding could be attributed to the fact that a rise in concentration raises the solution’s chroma, which influences the solution’s transmittance and prevents light from being absorbed on the 0.5M-ZnO@GAC catalyst surface [60]. Also, when the concentration of RhB increased, more RhB molecules were bonded to the 0.5M-ZnO@GAC surface, thereby lowering the active site for free radicals and decreasing the pace at which RhB degraded [13]. Although the rate of RhB degradation is rapid at low concentrations and the overall amount of RhB, degradation is high. However, when the concentration is high, the degradation efficiency is low [29,47].

The plot in Figure 5d and Table S3 shows the RhB degradation efficiency towards 0.5M-ZnO@GAC at varied catalyst doses (10, 30, 50, 70, and 90 mg/L and constant irradiation time of 120 min, pH 6, and RhB concentration of 5 mg/L). The results demonstrate that RhB degradation efficiency and photocatalytic rate constant (k_1_) under UV-A and UV-C lights increased with increasing amounts of 0.5M-ZnO@GAC catalyst. This could be ascribed to the fact that with increasing amounts of 0.5M-ZnO@GAC, more active sites and surface area are available, thereby promoting the formation of more radicals and superoxide, which resulted in higher RhB degradation [61,62,63]. 

### 3.3. Stability Test and TOC Removal

The ability of a catalytic material to be reused after different successive cycles is one way to determine its performance and stability for industrial applications. The dried 0.5 M-ZnO@GAC catalyst after the photodegradation experiment was reused for another five cycles under UV-A and UV-C lights, and the result is presented in Figure 5e. The first RhB degradation under UV-A and UV-C was 79.8% and 93.6%, respectively. The RhB degradation rates after the fifth cycle were 55.6% and 66.6%, suggesting an overall decrease rate from the first to the fifth cycle under UV-A and UV-C light of 24.2% and 27.4%, respectively. The decrease in the RhB degradation rate after each cycle could be due to catalyst blockage because of by-product formation during RhB degradation, leading to active site reduction [50]. The good efficiency of RhB degradation after the fifth cycle demonstrates the high stability and recyclability of 0.5M-ZnO@GAC in degrading RhB molecules under UV light.

The TOC removal of RhB degradation towards GAC, 0.25M-ZnO@GAC, 0.5M-ZnO@GAC, and 0.75M-ZnO@GAC at optimum conditions using 5 mg/L RhB concentration, a catalyst dosage of 50 mg/L, a pH of 6, and a photodegradation time of 120 min is depicted in Figure 5f. The TOC removal of GAC, 0.25M-ZnO@GAC, 0.5M-ZnO@GAC, and 0.75M-ZnO@GAC after 120 min were 11.5%, 33.2%, 79.1%, and 66.3%, respectively. The 0.5M-ZnO@GAC catalyst was seen to have the highest TOC removal rate. However, the calculated TOC rate for all the catalysts shows that the RhB mineralization efficiencies were below 100%, suggesting that some part of the RhB or its oxidized organic products was further oxidized into H_2_O and CO_2_ [44].

### 3.4. Adsorptive Performance

To examine the performance of GAC and the ZnO-based catalyst, the adsorptive capacity of the developed composites was tested towards RhB in batch adsorption study using varied contact time (0–120 min), RhB concentration of 50 mg/L, adsorbent dosage of 50 mg/L, and temperature of 298 K. The kinetic curves of q_e_ against t for GAC, 0.25M-ZnO@GAC, 0.5M-ZnO@GAC, and 0.75M-ZnO@GAC towards RhB adsorption are as shown in Figure S5a–d. The results revealed that a rapid adsorption rate was observed at an initial time of 30 min before the equilibrium position was achieved. However, the equilibrium position was achieved at 90 min for GAC while 0.25M-ZnO@GAC, 0.5M-ZnO@GAC, and 0.75M-ZnO@GAC attained equilibrium at 105 min. The adsorption capacity and RhB removal of GAC, 0.25M-ZnO@GAC, 0.5M-ZnO@GAC and 0.75M-ZnO@GAC towards RhB, shown in Figure S5e,f were 487.1 mg/g, 467.4 mg/g, 481.1 mg/g and 456.1 mg/g, resulting in 99.5%, 95.9%, 98.7%, and 93.8%, respectively. The adsorption capacity and RhB removal had an order of GAC > 0.5M-ZnO@GAC > 0.25M-ZnO@GAC > 0.75M-ZnO@GAC. The excellent adsorptive performance of GAC and ZnO@GAC towards RhB could be due to their larger BET surface area, appropriate pore diameter, moderate mesopore and micropore volume, and the abundant presence of O-containing functional groups on the materials’ surface [64]. The difference in the adsorption capacity and RhB removal could be ascribed to the pore structure of the materials. Furthermore, the decrease in the adsorption capacity and RhB removal towards 0.75M-ZnO@GAC could be because of micropore (V_micro_/V_total_= 53.6%) dominance, making it difficult for the RhB molecules to access some of the adsorbent active sites. However, the GAC, 0.25M-ZnO@GAC, 0.5M-ZnO@GAC possessed more mesopores (V_meso_/V_total_ = 72%, 59.3%, and 62.8%), which might promote the adsorption of RhB molecules.

(5) $$q_{t}{=q}_{e}{(1\;-\;\exp}^{k_{1}t}),$$

(6) $$q_{t}{=(q}_{e}^{2}k_{2}{t)/(1+q}_{e}k_{2}t),$$

where $q_{e}andq_{t}$ are the amount of RhB adsorbed at equilibrium and at any time, t; $k_{1}\;and\;k_{2}\;are$ the pseudo-first-order (min^−1^) and pseudo-second-order (g mg^−1^ min^−1^) rate constants; and t is adsorption time (min)

Also, the RhB adsorption data onto GAC, 0.25M-ZnO@GAC, 0.5M-ZnO@GAC and 0.75M-ZnO@GAC towards RhB which were fitted to non-linear form of pseudo-first order and pseudo-second order kinetic models (Equations (5) and (6)) demonstrate that the adsorption process followed pseudo-second-order kinetics based on high regression correlation (R^2^) values as shown in Table 4. This fact suggests that the adsorption process is majorly controlled by chemical adsorption [65]. Moreover, the pseudo-second-order kinetics experimental adsorption capacity (q_e, exp_) (487.1 mg/g, 467.4 mg/g, 481.1 mg/g, and 456.1 mg/g) values when compared with the pseudo-first-order were observed to be closer to the calculated adsorption capacity (q_e, cal_) (490.7 mg/g, 459.7 mg/g, 478.9 mg/g, and 449.9 mg/g) values for GAC, 0.25M-ZnO@GAC, 0.5M-ZnO@GAC and 0.75M-ZnO@GAC, respectively.

### 3.5. Plausible Photocatalytic Degradation Mechanism

In order to gain an understanding of the photocatalytic oxidation reaction and the mechanism of reaction between 0.5M-ZnO@GAC and RhB, the impact of active radicals on RhB degradation efficiency was investigated. The radical scavenger test was carried out using 0.005 M each of calcium persulfate (PS), isopropanol (IPA), triethanolamine (TEOA), and benzoquinone (BQ) to quench the hydroxyl radicals (^•^OH), superoxide radicals (O_2_^−•^), electrons (e^–^), and photoinduced holes (h^+^). The plot in Figure 5g demonstrated that addition of BQ and TEOA scavenger to the photocatalytic reaction resulted in a significant decrease in the RhB degradation efficiency under UV-A and UV-C lights from 87.89% and 97.56% to 43.34% and 51.66% for BQ while 32.56% and 37.97% was recorded for TEOA, suggesting the involvement of h^+^ and O_2_^−•^ in the photocatalytic activity process [65]. However, the slight decrease in RhB degradation efficiency under UV-A and UV-C lights (75.11% and 86.56%) in the presence of IPA, suggests that ^•^OH may have also played a role during the photocatalytic oxidation process [47,66]. Furthermore, it was concluded that e^–^ did not play a significant role during the oxidation process as a result of the minimal effect of PS under UV-A (<3%) and UV-C lights (<2%) [53]. 

In addition, the UV-vis spectra of the RhB solution during the photocatalytic process using GAC and ZnO-based composites as the catalysts under the influence of UV-A and UV-C lights were displayed in Figures S3a–e and S4a–e. It was obvious from the plots that the position of RhB’s maximum absorption peak (553 nm) gradually decreased in intensity as the irradiation time was extended and remained unchanged. On the other hand, the color of the RhB solution changed gradually from red to colorless, indicating that RhB’s structure had been damaged and degraded gradually into other by-products [67]. Furthermore, after exposure to UV-A and UV-C lights for 120 min, the RhB degradation efficiency was observed to be 82.42% and 97.11%, respectively.

**Figure 6:**
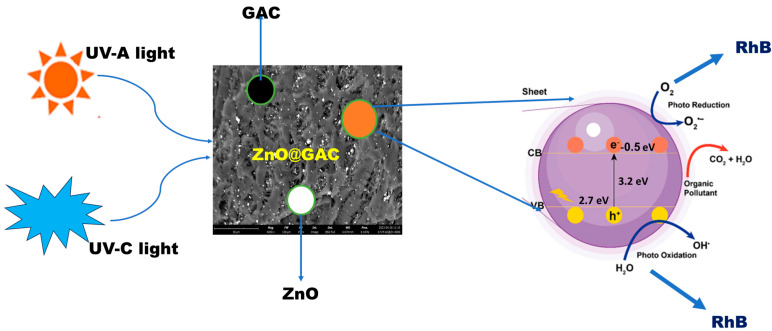
Proposed mechanism of reaction for RhB degradation under UV-A and UV-C lights.

The plausible mechanism of RhB photodegradation towards ZnO@GAC composites in a photocatalytic system is presented in Figure 6. The photodegradation mechanism of RhB takes place in an oxidation-reduction process. However, under the influence of UV-A and UV-C lights, electrons (e^–^) are transferred from the valence band (VB) to the conduction band (CB), resulting in the formation of positive charges (holes) in VB and negative charges (electron) in CB [29,68]. Furthermore, during the photocatalytic process, the oxidation of RhB molecules was initiated by the VB-holes and this could interact with the OH^−^ to produce hydroxyl radicals or oxidize the pollutants directly [69]. Again, the CB electrons can form reactive oxygen species (ROS) when they are transferred to dissolved O_2_ molecules or OH^−^. As a result, RhB is degraded because of the reaction’s photoactive radicals, which could form CO_2_, H_2_O, and other intermediates [70,71]. Thus, it can be suggested that the RhB photocatalytic process involves photogenerated holes in VB and O_2_^−•^ [53,60,72]. The photocatalytic oxidation mechanism of RhB can be expressed using the equations:

(7) $$ZnO@GAC+hv\;\left(UV-a\;and\;UV-c\;lights\right)\to ZnO@GAC\;(e^{-}+h^{+})$$

(8) $${OH}^{-}+h^{+}\to ∙OH$$

(9) $$e^{-}+O_{2}\to O_{2}^{-∙}$$

(10) $$RhB+h^{+}/O_{2}^{-∙}\to chromophore\;structure\;damage\to {CO}_{2}+H_{2}O$$

However, the RhB degradation efficiency using 0.5M-ZnO@GAC was compared with other catalysts in the literature, and the findings are presented in Table 5. The results showed that the present study exhibited an outstanding performance towards RhB degradation.

**Figure 7:**
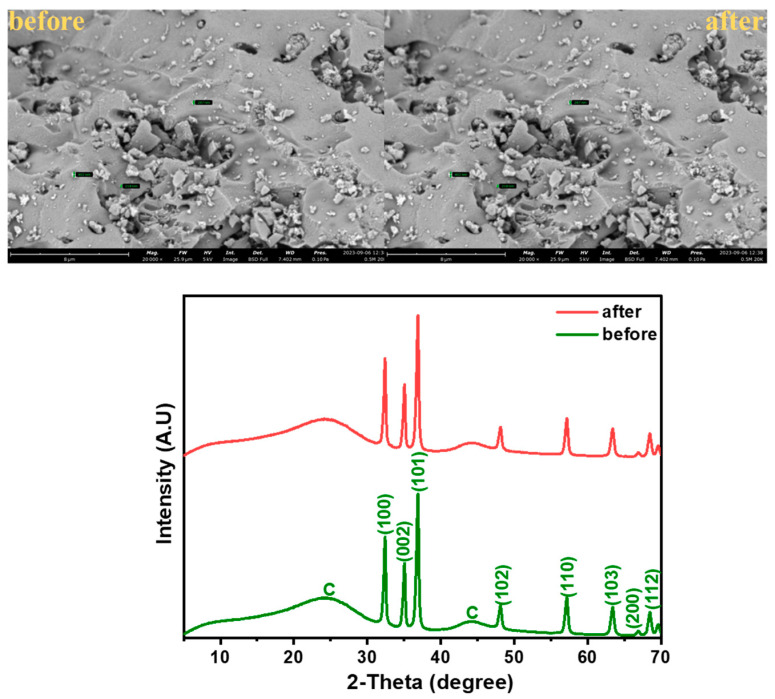
SEM micrographs and XRD patterns of 0.5M-ZnO@GAC before and after adsorption-photocatalytic degradation.

Moreover, the SEM and XRD analysis were examined on the 0.5M-ZnO@GAC catalyst material before and after RhB photocatalytic activity, as depicted in Figure 7. The results observed that there was no noticeable change in the SEM images and XRD patterns before and after the photocatalytic reaction. The SEM structure and XRD crystalline nature after photocatalytic degradation were not damaged. Also, the XRD pattern shows that no peak was added, shifted, or disappeared after degradation [55,73,74]. These observations suggest that the developed catalysts retain their structural and crystalline nature and also demonstrate excellent chemical stability and photostat stability after photodegradation.

**Table 5:** Comparative studies of RhB degradation efficiency with various catalysts reported in literature.

Catalyst	Irradiation Time (min)	Light Source	PDE (%)	References
PTh/Ag_3_PO_4_/BiOI/Ti– Cu_2_O/Cu	120	Visible light	96	[75]
MTiO_3_@EDFG	120	Visible light	91	[53]
SnFe_2_O_4_/Bi_2_WO_6_	120	350-W xenon lamp	96	[54]
H [K_2_Ag_9_(DPT)_7_ (u_-2_-O)_2_(H_2_O)_4_][SiW_12_O_40_]_2_	300	UV light radiation	76	[76]
AgNPs@ZnO	180	500 W Xe lamp	95	[77]
Zn_0.5_Mn_0.5_Ce_0.08_Fe_1.92_O_4_	180	Visible light	97	[78]
TiO_2_/rGO (5%)	120	Visible light	95	[79]
Co_3_O_4_/ZnFe_2_O_4_	240	UV light	93	[80]
ZnO: Mo/rGO films	120	Sunlight	68	[81]
MoS_2_/PMS	120	Visible light	90	[66]
0.5M-ZnO@GAC	120	UV-A light	82	This study
0.5M-ZnO@GAC	120	UV-C light	97	This study

## 4. Conclusions

In summary, different concentrations of Zn solution (0.25 M, 0.5 M, and 0.75 M) were used to load ZnO onto GAC via an in-situ method to develop 0.25M-ZnO@GAC, 0.5M-ZnO@GAC, and 0.75M-ZnO@GAC for the collective adsorption and photocatalytic degradation of RhB. The multifunctional ZnO is more effective as a semiconducting photocatalyst because it is cheap, environmentally benign, structure-dependent, and has the ability to mineralize pollutants completely. In comparison with GAC, 0.25M-ZnO@GAC, and 0.75M-ZnO@GAC, the 0.5M-ZnO@GAC showed excellent photocatalytic activity in RhB degradation under UV-A and UV-C lights due to their larger surface area, ability to absorb light, separation efficiency, and transfer of the photogenerated charge carrier. The photocatalytic degradation efficiency of RhB towards 0.5M-ZnO@GAC under UV-A and UV-C lights reached 82% and 97% within 120 min with rate constants of 0.010 min^−1^ and 0.019 min^−1^, respectively. The scavenger experiments demonstrated that hydroxyl (^•^OH), superoxide (O_2_^−•^), and photoinduced holes (h^+^) are the primary reactive radicals in the photocatalytic system. Furthermore, 0.5M-ZnO@GAC photocatalyst exhibited high stability with RhB degradation efficiency of 55.6% and 66.2% after five degradation cycles under UV-A and UV-C lights, respectively. The TOC experiment conducted revealed that the RhB mineralization rate towards 0.5M-ZnO@GAC reached 79.1%. The present study also proposed a plausible photocatalytic mechanism of reaction for RhB and 0.5M-ZnO@GAC interactions in the presence of UV light with reactive oxygen species playing a key role. In a batch adsorption system, RhB adsorption towards GAC exhibited the highest adsorption capacity (487.1 mg/g) and RhB removal (99.5%) with fast adsorption kinetics within 90 min. Furthermore, the pseudo-second-order kinetic model could explain the spontaneous RhB adsorption towards GAC. Finally, the findings herein offer new perspectives for developing innovative and effective photocatalysts for RhB adsorption and photodegradation under UV-A and UV-C lights in an aqueous medium.

## Figures and Tables

**Table 1:** Textural properties of developed GAC- and ZnO-based nanocomposites.

Properties		Materials		
	GAC	0.25M-ZnO@GAC	0.5M-ZnO@GAC	0.75M-ZnO@GAC
S_BET_ (m^2^/g)	474	450	453	421
S_Lang_ (m^2^/g)	707	675	679	632
S_mic_ (m^2^/g)	325	288	298	245
S_mic_/S_BET_ (%)	68.57	64.0	65.78	58.19
S_ext_ (m^2^/g)	149	162	155	176
S_ext_/S_BET_ (%)	31.43	36.0	34.22	41.81
V_tot_ (cm^3^/g)	0.2683	0.2727	0.2659	0.2620
V_meso_ (cm^3^/g)	0.1931	0.1618	0.1670	0.1217
V_mic_ (cm^3^/g)	0.0752	0.1109	0.0989	0.1403
V_meso_/V_tot_ (%)	71.97	59.33	62.81	46.45
V_mic_/V_tot_ (%)	28.03	40.67	37.19	53.55
D_p_ (nm)	2.26	2.42	2.35	2.49

BET surface area (S_BET_), Langmuir surface area (S_Lang_), micropore surface area (S_mic_), external surface area (S_ext_), total pore volume (V_tot_), micropore pore volume (V_mic_), and pore size (D_p_).

**Table 2:** Elemental composition GAC and GAC-ZnO-based composites at different spots.

Sample	Particle 1			Particle 2			Particle 3		
	Weight (%)			Weight (%)			Weight (%)		
	C	O	Zn	C	O	Zn	C	O	Zn
GAC	92.6	7.2	-	92.0	9.1	-	86.4	10.4	-
0.25M-ZnO@GAC	43.0	5.9	17.3	51.5	8.6	3.2	68.6	3.8	3.6
0.5M-ZnO@GAC	23.5	15.5	12.5	47.1	7.5	15.9	42.0	7.2	8.9
0.75M-ZnO@GAC	22.7	11.6	30.6	21.8	12.2	29.2	39.5	10.0	20.2

**Table 3:** Pseudo-first-order kinetic parameters for RhB, GAC, and ZnO-based composites.

Materials	UV-A Light		UV-C Light	
	PDE (%)	k_1_ (min^−1^)	PDE (%)	k_1_ (min^−1^)
RhB	5.30	0.00028	10.12	0.00071
GAC	42.33	0.0025	57.12	0.0051
0.25M-ZnO@GAC	72.09	0.0079	80.37	0.097
0.5M-ZnO@GAC	82.42	0.010	97.11	0.019
0.75M-ZnO@GAC	70.98	0.0078	78.58	0.0089

PDE = photocatalytic degradation efficiency, and k_1_ = photocatalytic degradation rate constant.

**Table 4:** Adsorption kinetic parameters.

Sample	Pseudo-First-Order			Pseudo-Second-Order		
	k_1_ (min^−1^)	q_e_ (mg/g)	R^2^	k_2_ (g/(mg.min)	q_e_ (mg/g)	R^2^
GAC	0.010	668.70	0.992	0.0000053	490.67	0.999
0.25M-ZnO@GAC	0.021	549.77	0.984	0.000024	459.72	0.999
0.5M-ZnO@GAC	0.013	617.28	0.994	0.0000086	478.91	0.999
0.75M-ZnO@GAC	0.0085	730.55	0.990	0.0000045	449.88	0.990

## Data Availability

Data will be made available on request.

## Supplementary Materials

The following supporting information can be downloaded at: https://www.mdpi.com/article/10.3390/nano14141234/s1.
