# Supplementary material for: In-Situ Hydrothermal Fabrication of ZnO-Loaded GAC Nanocomposite for Efficient Rhodamine B Dye Removal via Synergistic Photocatalytic and Adsorptive Performance
Source: Nanomaterials (Basel). 2024 Jul 22;14(14):1234. doi: 10.3390/nano14141234 (PMC11279918; doi:10.3390/nano14141234)
Supplement: Supplementary file 1 [file nanomaterials-14-01234-s001.zip › nanomaterials-3079995-supplementary.pdf]

nanomaterials

MDPI

Supplementary Materials

In-situ Hydrothermal Fabrication of ZnO-Loaded GAC Nanocomposite for Efficient Rhodamine B Dye Removal via Synergistic Photocatalytic and Adsorptive Performance

Kehinde Shola Obayomi [1,2,*], Sie Yon Lau [1], Zongli Xie [3], Stephen R. Gray [2] and Jianhua Zhang [2,*]

1 Department of Chemical Engineering, Curtin University, CDT 250, 98009 Miri, Sarawak, Malaysia
2 Institute for Sustainable Industries and Liveable Cities, Victoria University, Werribee, VIC 3030, Australia
3 Commonwealth Scientific and Industrial Research Organization (CSIRO), Private Bag 10, Clayton South, Vic. 3169, Australia
* Correspondence: obayomikehindeshola@gmail.com (K.S.O.); jianhua.zhang@vu.edu.au (J.Z.)

Table S1. Pseudo-first order kinetic parameters for RhB degradation on 0.5M-ZnO@GAC in different pH solution under UV-A and UV-C lights.

pH	UV-A light		UV-C light	
	PDE (%)	k_1 (min^{-1})	PDE (%)	k_1 (min^{-1})
2	73.27	0.041	80.32	0.088
4	81.32	0.074	89.64	0.097
6	88.76	0.091	99.34	0.121
8	80.63	0.066	94.63	0.101
10	75.32	0.054	90.26	0.076

Table S2. Pseudo-first order kinetic parameters for RhB degradation on 0.5M-ZnO@GAC in different initial RhB concentrations under UV-a and UV-C lights.

RhB concentration (mg/L)	UV-A light		UV-C light	
	PDE (%)	k_1 (min^{-1})	PDE (%)	k_1 (min^{-1})
5	90.32	0.091	99.24	0.124
10	82.42	0.085	97.11	0.117
15	77.32	0.056	93.46	0.102
20	70.24	0.031	89.32	0.088
25	66.34	0.023	82.45	0.061

Table S3. Pseudo-first order kinetic parameters for RhB degradation on 0.5M-ZnO@GAC in different 0.5M-ZnO@GAC dosage under UV-A and UV-C lights.

Catalyst (mg/L)	UV-A light		UV-C light	
	PDE (%)	k_1 (min^{-1})	PDE (%)	k_1 (min^{-1})
10	69.25	0.0045	81.02	0.056
30	73.32	0.0081	87.42	0.099
50	85.86	0.0096	97.32	0.101
70	89.34	0.064	98.63	0.109
90	93.26	0.024	99.67	0.129

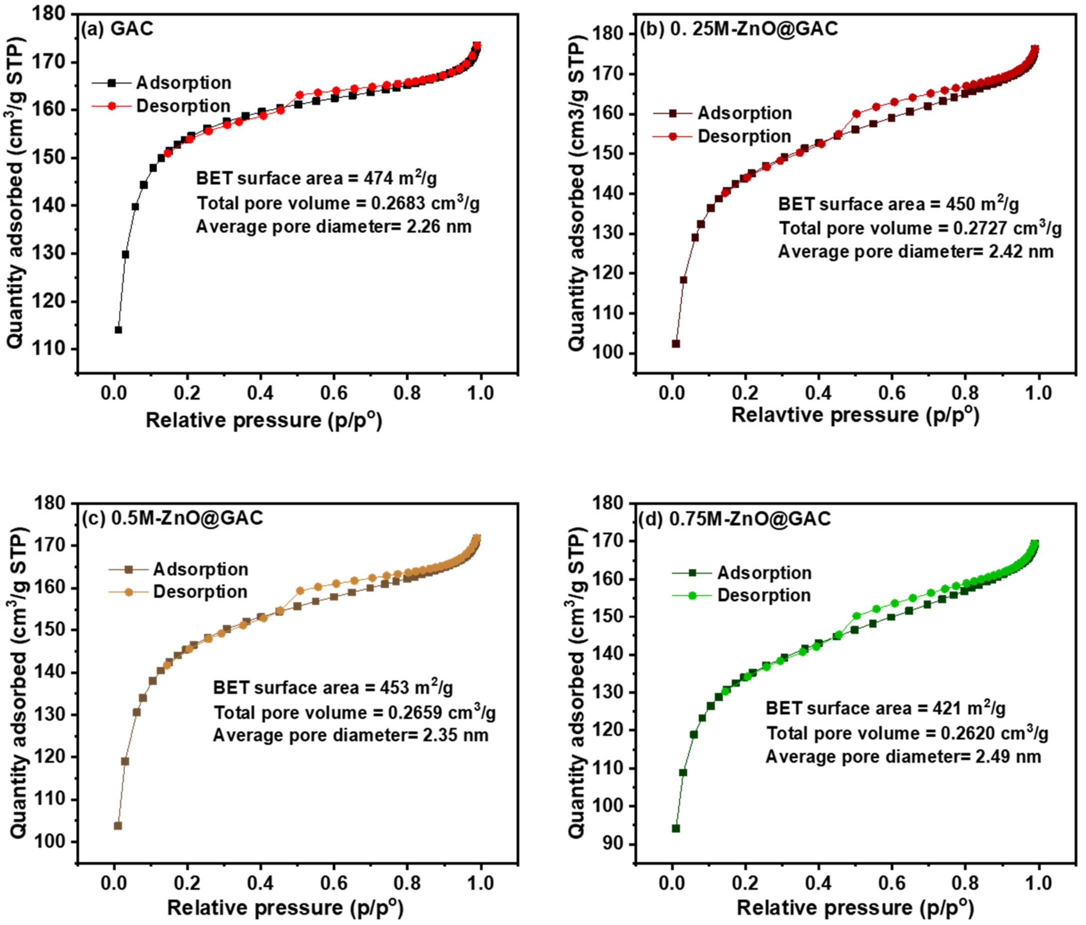

Figure S1. N_2 adsorption-desorption curve for (**a**) GAC, (**b**) 0.25M-ZnO@GAC, (**c**) 0.5M-ZnO@GAC, and (**d**) 0.75M-ZnO@GAC.

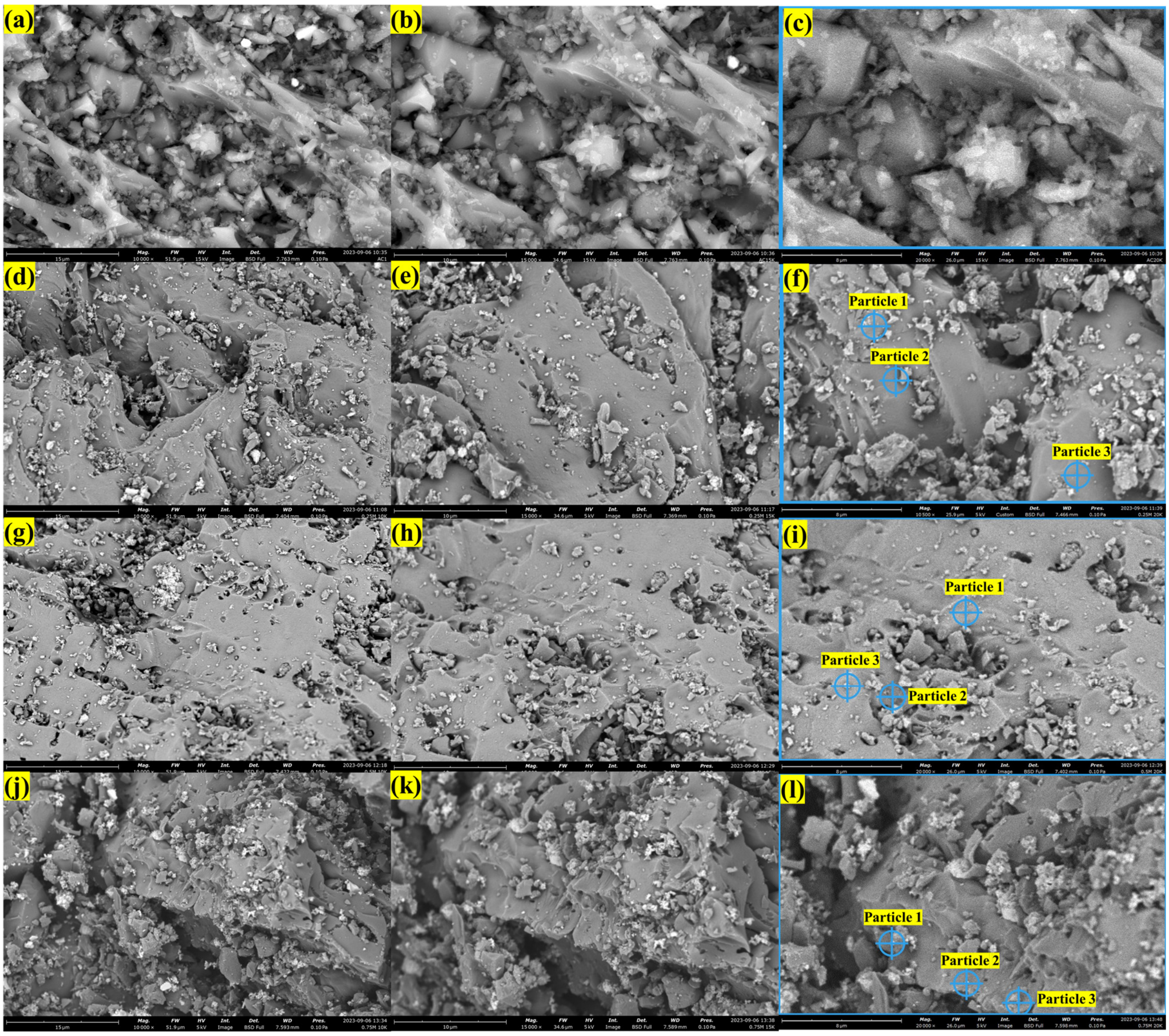

Figure S2. SEM micrographs at different magnification with mark spot to show the presence of ZnO for GAC (**a–c**), 0.25M-ZnO@GAC (**d–f**), 0.25M-ZnO@GAC (**g–i**), and 0.25M-ZnO@GAC (**j–l**).

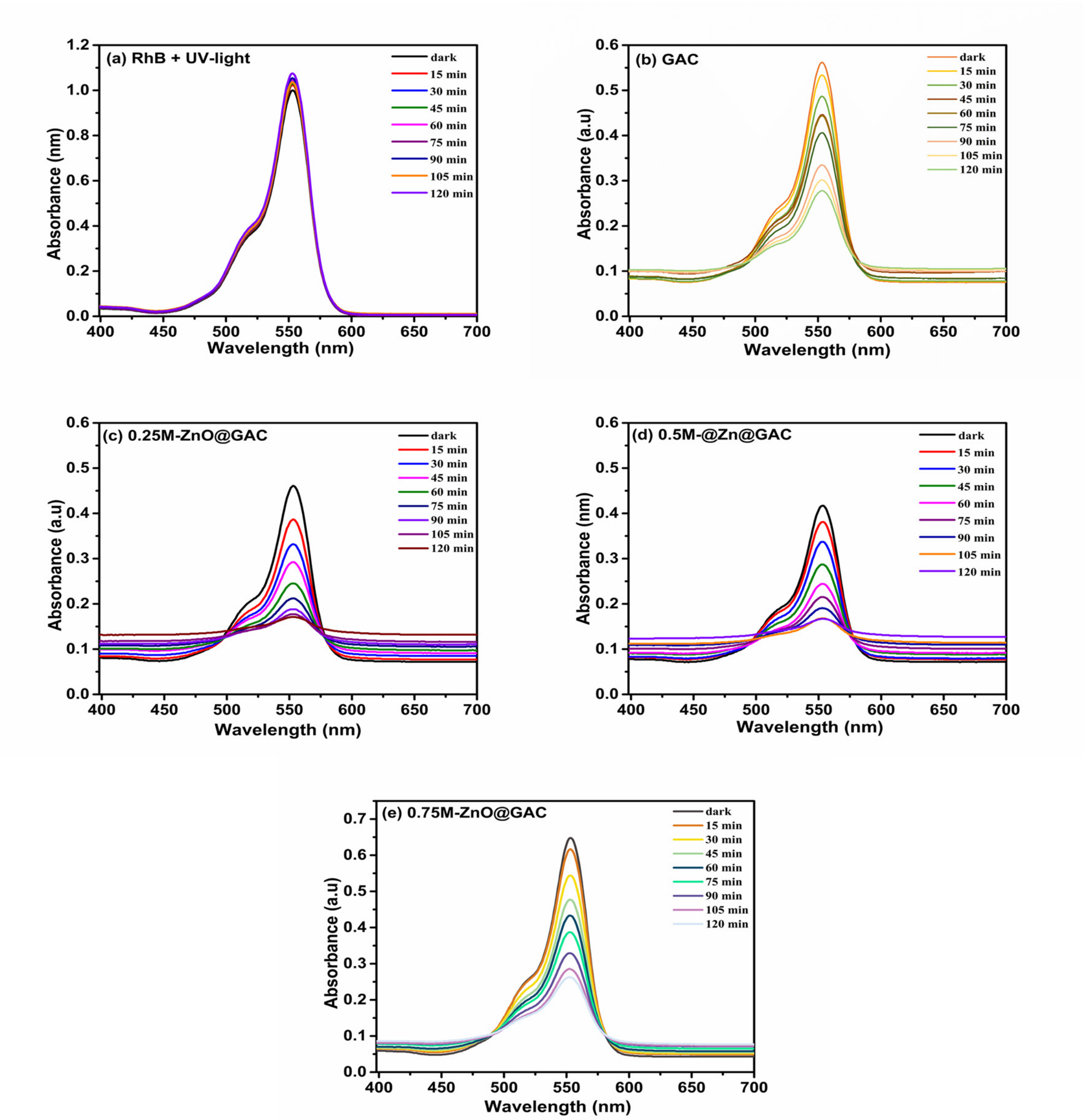

Figure S3. (**a**–**f**) UV–Vis time-dependent spectra of (**a**) RhB, (**b**) GAC, (**c**) 0.25M-ZnO@GAC, (**d**) 0.5M-ZnO@GAC, and (**e**) 0.75M-ZnO@GAC under UV-A light (initial RhB pH, initial concentration= 5 mg/L and temperature =298 K).

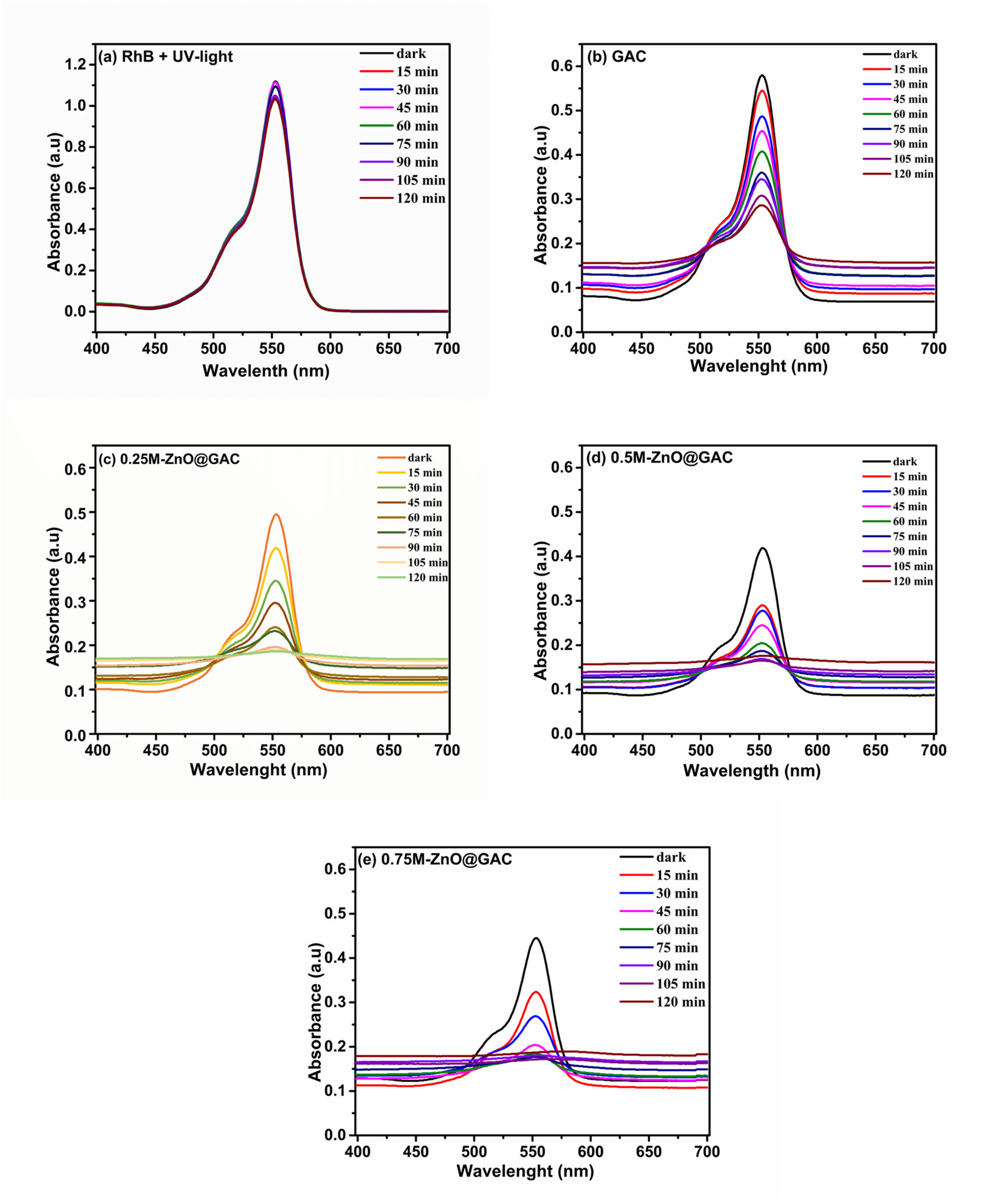

Figure S4. (a–f). UV–Vis time-dependent spectra of (a) RhB (b) GAC, (c) 0.25M-ZnO-GAC, (d) 0.5M-ZnO-GAC, and (e) 0.75M-ZnO-GAC under UV-C light (initial RhB pH, initial concentration= 5 mg/L and temperature =298 K).

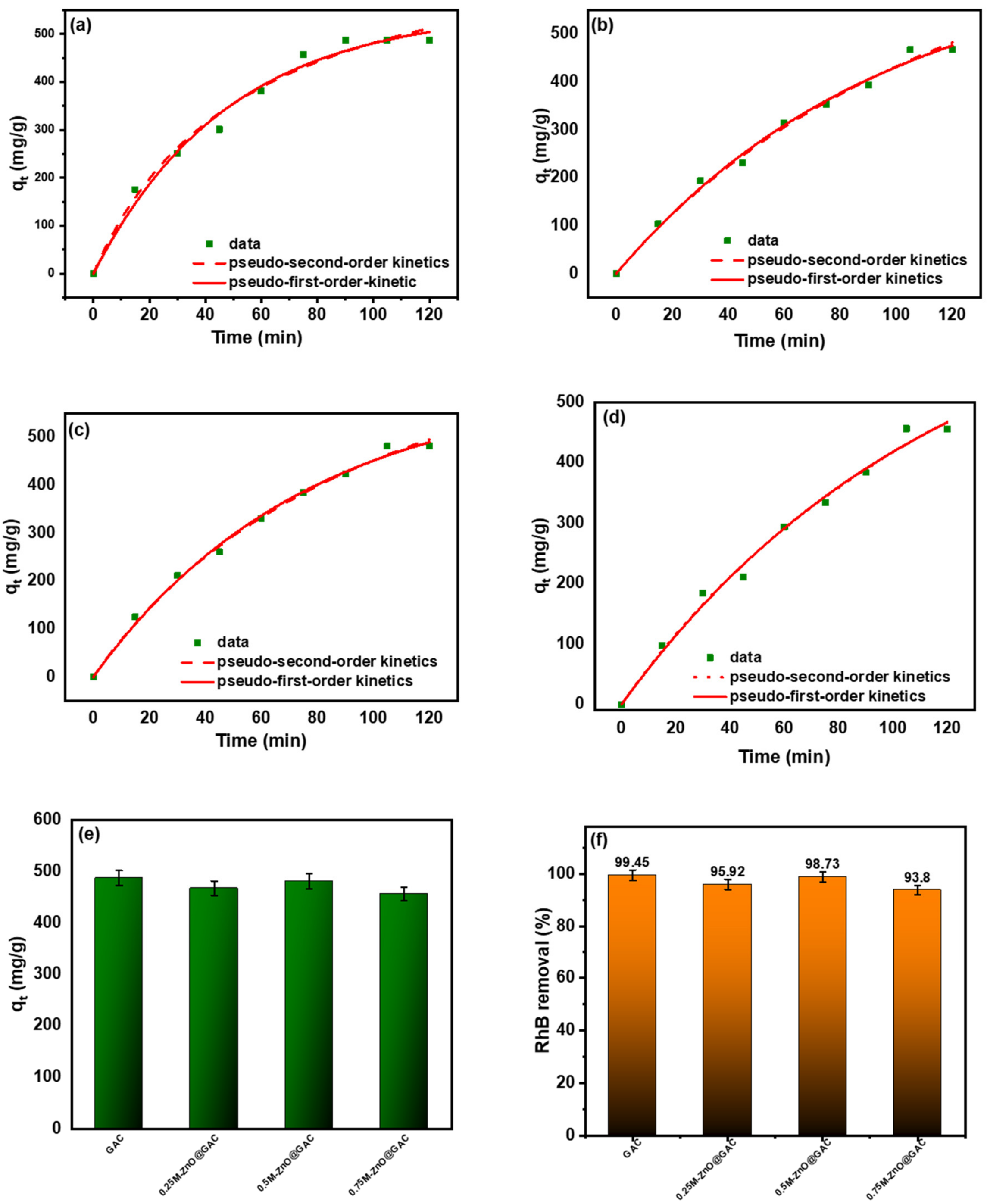

Figure S5. Plots of (**a**–**d**) adsorption kinetics, (**e**) adsorption capacity, and (**f**) RhB removal of GAC, 0.25M-ZnO@GAC, 0.5M-ZnO@GAC, and 0.75M-ZnO@GAC towards RhB.
